# Chemoinformatic Analysis as a Tool for Prioritization of Trypanocidal Marine Derived Lead Compounds 

**DOI:** 10.3390/md12031169

**Published:** 2014-03-04

**Authors:** Yunjiang Feng, Marc Campitelli, Rohan A. Davis, Ronald J. Quinn

**Affiliations:** Eskitis Institute for Drug Discovery, Griffith University, Brisbane, QLD 4111, Australia; E-Mails: y.feng@griffith.edu.au (Y.F.); marc.campitelli@gmail.com (M.C.); r.davis@griffith.edu.au (R.A.D.)

**Keywords:** trypanosomiasis, marine natural products, chemical clustering, ChemGPS-NP, drug-like physicochemical properties

## Abstract

Marine trypanocidal natural products are, most often, reported with trypanocidal activity and selectivity against human cell lines. The triaging of hits requires a consideration of chemical tractability for drug development. We utilized a combined Lipinski’s rule-of-five, chemical clustering and ChemGPS-NP principle analysis to analyze a set of 40 antitrypanosomal natural products for their drug like properties and chemical space. The analyses identified 16 chemical clusters with 11 well positioned within drug-like chemical space. This study demonstrated that our combined analysis can be used as an important strategy for prioritization of active marine natural products for further investigation.

## 1. Introduction

Human African Trypanosomiasis (HAT), also known as African Sleeping Sickness, is a fatal disease transmitted by two species of a protozoan parasite, *T. brucei rhodesiense* and *T. brucei gambiense*. *T. b. rhodesiense* is the agent of the acute form of the disease, prevailing in Eastern and Southern Africa and *T. b. gambiense* causes the chronic form of the disease in Western and Central Africa. According to the latest figures from the World Health Organization (WHO), African Sleeping Sickness threatens 70 million people in resource-poor regions of Africa, and is the world’s third most devastating parasite disease [1]. Since the disease predominantly afflicts the very poor, it is designated as a neglected tropical disease. The registered drugs suramin and pentamidine are not effective against both stages of the disease. The second stage effective drug melarsoprol has associated toxicity which has been reported as lethal in up to 12% of cases [2]. There have also been reports of incidence of drug resistance in HAT cases [2,3]. There is an urgent need for the development of new, safer and more effective drugs to fight African Sleeping Sickness. 

The search for antitrypanosomal agents has predominantly focused on synthetic efforts. A series of purine nitriles synthesized by combinational chemistry have showed potent trypanocidal activity and a high degree of selectivity [4]. Most recently, Sanofi-Aventis and Drugs for Neglected Diseases initiative (DNDi) have announced an agreement for the development, manufacturing and distribution of fexinidazole, a promising new drug for the treatment of African Sleeping Sickness [5]. Though natural product research has not played a central role in the search for antitrypanosomal therapeutics, there are emerging numbers of compounds from plants and marine organisms with promising activity against trypanosomiasis [6,7,8].

We have previously reported a series of marine natural products active against *T. b. brucei* [9,10,11,12,13]. In most cases, natural products research stops when new structures and their associated biological activities are published. We wish to develop a strategy to prioritize these molecules for further investigation. We conducted further analysis to evaluate the drug-like properties and chemical space of these and other compounds. In this paper, we will discuss the chemoinformatic methods we used to conduct the analysis, including Lipinski’s rule-of-five, chemical clustering and ChemGPS-NP principle component analysis, as well as the results of these analyses. 

## 2. Results and Discussion

**Figure 1:**
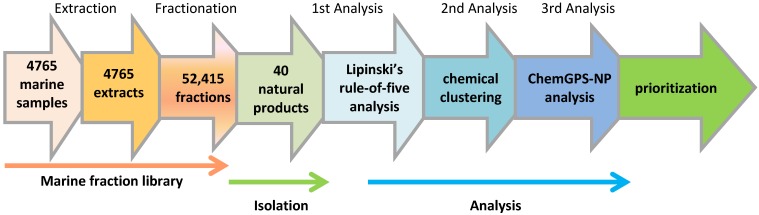
An overview of the natural product discovery program including the construction of the marine fraction library, the isolation of bioactive natural products, and rule-of-five, chemical clustering and ChemGPS-NP principle component analysis for prioritization.

The overall outline of the natural product discovery program is shown in Figure 1. The objective was to front-load both crude extracts and subsequent fractions with desirable physicochemical properties, rapidly isolate natural products that are principally located within biologically relevant chemical space, and prioritize isolated compounds for further chemical and biological investigation. 

### 2.1. Marine Fraction Library

**Figure 2:**
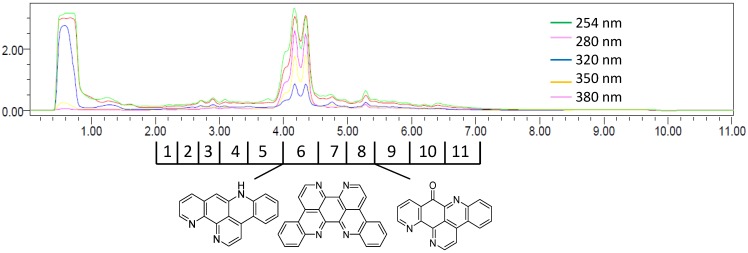
An example of a lead-like enhanced extract HPLC chromatogram. Three active pyridoacridine alkaloids were isolated from the fractions 6–8 [10].

A pre-fractionated library was constructed using a proprietary lead-like enhanced extraction and fractionation protocol developed in-house [14,15]. The crude CH_2_Cl_2_ and MeOH extracts from Australian marine organisms were first loaded onto solid-phase absorbent poly(divinylbenzene-*N*-vinylpyrrolidone) copolymer (Waters Oasis HLB) eluting with MeOH/H_2_O (70:30) containing 1% trifluoroacetic acid (TFA). The MeOH/H_2_O (70:30) eluent which was proven to contain constituents with calculated log *P* < 5. The fraction library was constructed using reverse-phase solvent conditions (MeOH/H_2_O/0.1% TFA) on a C_18_ Monolithic HPLC column. Eleven fractions were collected per extract between 2 and 7 min of the chromatogram where constituents had calculated log *P* < 5 (Figure 2). The fractionation provided a second filtration of log *P* allowing constituents with high log *P* to be removed. The fractionation process also separated the complex crude extracts into fractions containing a small number of compounds to facilitate the rapid identification of active molecules. 

4765 Australian marine organisms were extracted and fractionated to construct the marine library consisting of 52,415 fractions. These marine organisms represented over 200 families and 420 genera. The organisms were collected from tropical and sub-tropical Queensland and temperate Tasmanian waters in Australia.

### 2.2. Taxonomic Origin

**Figure 3:**
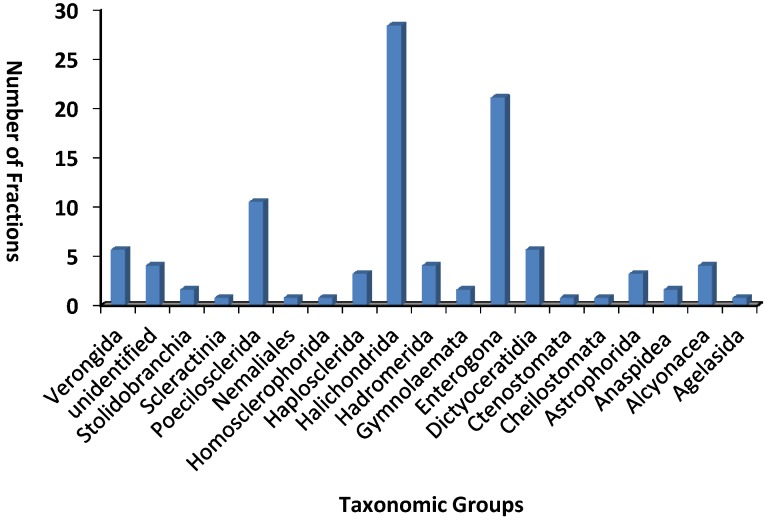
Taxonomic clusters of the 83 marine fractions. The fractions represented 18 unique orders of marine organisms with Halichondrida (28 samples), Enterogona (21 samples) and Poecilosclerida (10 samples) most abundant. Four fractions were from unidentified marine organisms.

**Figure 4:**
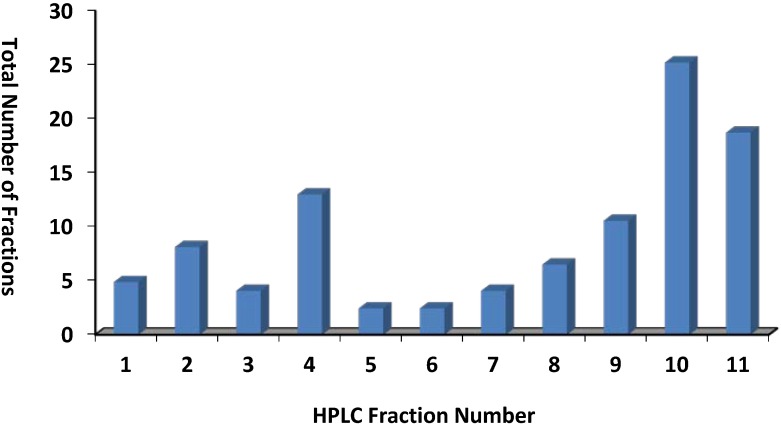
Number of fractions distributed in HPLC chromatograms. The fractions relatively evenly distributed within 11 HPLC fractions with 45 in fractions 10 and 11.

Eighty three marine fractions were identified as active against *T. b. brucei* and chemically investigated. The 83 fractions belonged to 18 different orders and 50 different families, representing sponges (Verongida, Poecilosclerida, Homosclerophorida, Haplosclerida, Halichondria, Hadromerida, Dictyoceratidia, Astrophorida and Agelasida), ascidians (Stolidobranchia and Entergona), cnidarians (Scleractinia and Alcyonacea), bryozoans (Gymnolaemata, Ctenostomata and Cheilostomata), algae (Nemaliales) and mollusk (Anaspidea). Four fractions were from unidentified marine organisms. The three most represented taxonomic orders were Halichondrida (28 samples), Enterogona (21 samples) and Poecilosclerida (10 samples) (Figure 3). Further analysis showed that the fractions were distributed across all 11 fractions, with more than half originating from the relatively nonpolar fractions 10 and 11 (Figure 4).

### 2.3. Isolation of Marine Natural Products

A preliminary chemical analysis was conducted on the 83 active fractions using LC-MS and ^1^H-NMR spectroscopy. Based on the MS and ^1^H-NMR data, two de-replication processes were carried out. The first process was the identification of the same compounds in multiple fractions of the same biota. In this case, the isolation was carried out on only one of the fractions. The second process was the recognition of the same compounds in fractions of different biota. The different biota was then classified into one cluster, and the isolation and purification was carried out on one of the biota from the cluster. 

**Figure 5:**
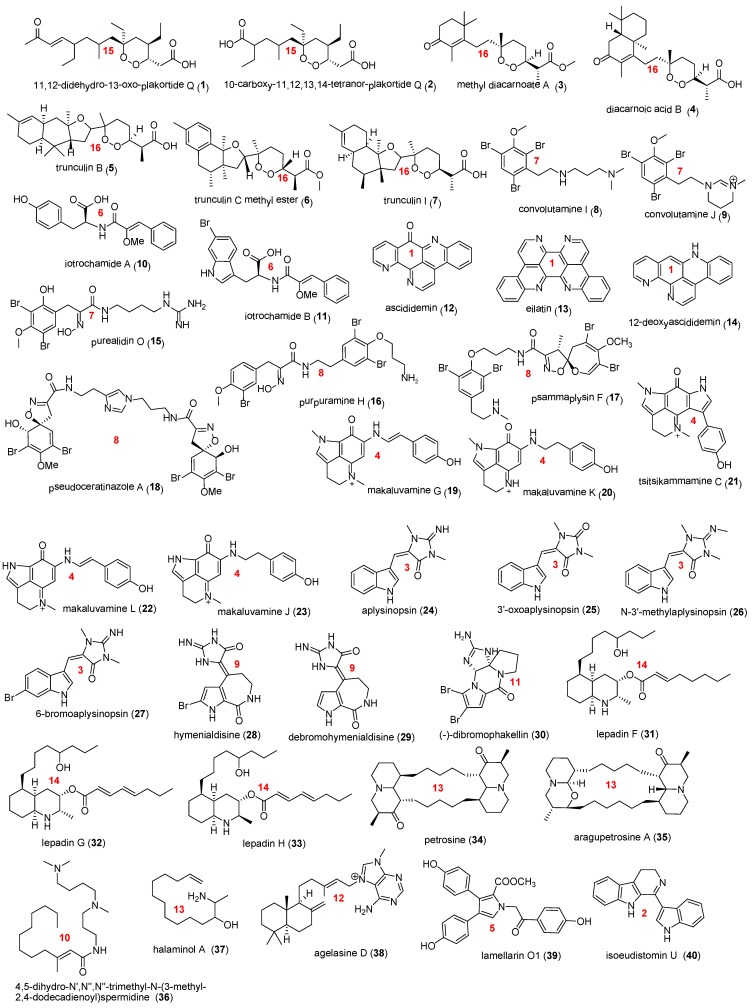
Chemical structures of the 40 marine natural products; the number in red represents their unique chemical cluster.

Ten grams of freeze-dried and ground marine samples were extracted with CH_2_Cl_2_ and MeOH, the combined extracts were purified using a targeted isolation procedure. This procedure was based on the HPLC retention time, UV chromophore, mass and ^1^H NMR spectroscopic data. Purification of natural products was achieved predominantly by C_18_ bonded silica HPLC eluting with gradients using MeOH/H_2_O containing 0.1% TFA. This has been discussed in details in previous publications [9,11,12]. A total of 40 pure natural products (Figure 5) were isolated, including cyclic peroxides (**1**–**7**) [11,16,17,18,19], aryl amines (**8**–**9**) [13], cinnamoyl amino acids (**10**–**11**) [9], pyridoacridine alkaloids (**12**–**14**) [10], bromotyrosine derivatives (**15**–**18**) [12,20,21], makaluvamine alkaloids (**19**–**23**) [22,23,24], aplysinopsins (**24**–**27**) [25,26], hymenialdisine alkaloids (**28**–**30**) [27,28], lepadins (**31**–**33**) [29,30], petrosine alkaloids (**34**–**35**) [31,32,33], amino hydrocarbons (**36**–**37**) [34,35], a purine derivative (**38**) [36], a pyrrole alkaloid (**39**) [37], and a β-carboline alkaloid (**40**) [38]. 

### 2.4. Antitrypanosomal Activities

Cyclic peroxides were one of the largest, most potent, and chemically unique chemotypes identified [3]. 11,12-Didehydro-13-oxo-plakortide Q (**1**) exhibited potent activity against *T. b. brucei* with an IC_50_ value of 49 nM [3]. 10-carboxy-11,12,13,14-tetranor-plakortide Q (**2**), where the enone functional group was replaced with a carboxylic acid group in the side chain, had reduced activity with an IC_50_ of 940 nM [3]. Preliminary cytotoxicity study indicated that **1** had an IC_50_ value of 5.1 µM against the human embryonic kidney cell line HEK293, showing a 105-fold selectivity, while **2** showed 100% inhibition of HEK293 at 83 µM [3]. A number of analogues were synthesised based on the natural product scaffold [39].

The aryl amine derivatives from the broyozoan *Amathia tortusa*, convolutamine I (**8**) and J (**9**), were also shown to be active against *T. b. brucei* with IC_50_ values of 1.1 and 13.7 μM, respectively [13]. Preliminary toxicity profiling suggested that convolutamine I (**8**) exhibited cytotoxicity against HEK293 with an IC_50_ of 22.0 μM whilst convolutamine J (**9**) was inactive at 41.0 μM [13].

The cinnamoyl amino acids, iotrochamides A (**10**) and B (**11**), both exhibited activity against *T. b. brucei* with IC_50_ values of 4.7 and 3.4 µM, respectively [9]. Compounds **10** and **11** also had 85% inhibition at 50 µM and 100% inhibition at 70 µM against HEK293, respectively [9]. These results indicated that iotrochamides A (**10**) and B (**11**) showed some moderate selectivity towards *T. b. brucei* [9].

The pyridoacridine derivatives, ascididemin (**12**), eilatin (**13**) and 12-deoxyascididemin (**14**), all exhibited potent activity against *T. b. brucei* with IC_50_ values of 0.032, 1.33 and 0.077 µM, respectively [10]. The compounds (**12**) and (**14**) also showed a 46- and 99-fold selectivity toward *T. b. brucei*. Compound (**13)** exhibited a plateau of 62% inhibition against HEK293 at the top three screening concentrations (21, 42 and 83 µM) [10].

Pseudoceratinazol A (**18**), a novel bromotyrosine derivative isolated from Australian marine sponge *Pseudoceratina* sp., had moderate antitrypanosomal activity with 80% inhibition of *T. b. brucei* at 83 µM [4].

### 2.5. Lipinski’s Rule-of-Five

**Figure 6:**
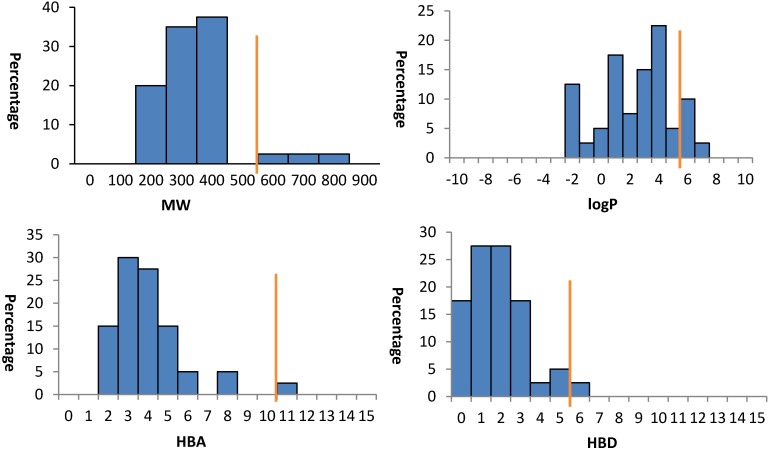
Analysis of physicochemical properties (MW, log *P*, HBA and HBD) of 40 isolated natural products. In each case the orange line indicates the maximum desirable value for oral bioavailability defined by Lipinski’s rule-of-five: MW < 500 Da; log *P* < 5, HBA < 10 and HBD < 5.

The drug- and lead-like physical and chemical properties of these natural products were calculated using Instant JChem (version 6.03) [40]. The parameters including molecular weight (MW), log *P*, number of hydrogen bond acceptors (HBA), and number of hydrogen bond donors (HBD) were analysed against Lipinski’s rule-of-five (Table 1 and Figure 6).

**Table 1:** Physicochemical profiling of isolated natural products **1**–**40**.

Compound	MW	Log *D*_5.5_	Log *P*	HBA	HBD	%PSA	No. of Violations
**1**	368.51	4.37	5.29	5	1	11.28	1
**2**	344.44	2.40	4.33	6	2	15.65	0
**3**	352.47	4.20	4.20	4	0	10.28	0
**4**	406.56	4.12	5.36	5	1	10.54	1
**5**	406.56	3.30	4.82	5	1	9.58	0
**6**	416.55	5.60	5.60	4	0	7.76	1
**7**	406.56	3.37	4.98	5	1	9.60	0
**8**	473.04	−1.64	4.05	3	1	4.88	0
**9**	470.02	0.29	0.29	2	0	3.37	0
**10**	341.36	0.76	2.55	5	3	20.20	0
**11**	443.29	1.61	3.72	4	3	17.46	0
**12**	283.28	2.98	2.98	4	0	16.07	0
**13**	356.38	4.70	4.70	4	0	11.78	0
**14**	269.30	3.15	3.40	3	1	10.92	0
**15**	495.17	0.66	1.31	8	6	28.51	1
**16**	622.15	2.85	3.57	6	3	16.26	1
**17**	747.07	0.65	3.74	8	3	15.77	1
**18**	898.19	−0.09	0.62	11	4	21.16	2
**19**	334.39	−1.43	−1.44	3	2	12.63	0
**20**	321.37	−0.94	1.82	4	2	14.92	0
**21**	332.38	−0.75	−0.75	2	2	13.52	0
**22**	320.37	−1.65	−1.67	3	3	16.28	0
**23**	322.38	−1.76	−1.76	3	3	15.17	0
**24**	254.29	1.39	1.39	3	2	18.44	0
**25**	255.27	1.22	1.22	2	1	16.58	0
**26**	268.31	1.61	1.61	3	1	13.69	0
**27**	333.18	2.15	2.15	3	2	17.44	0
**28**	324.13	−0.83	−0.83	4	5	35.30	0
**29**	245.24	−1.30	−1.30	4	5	37.79	0
**30**	389.05	−0.80	1.55	4	2	21.89	0
**31**	421.66	3.69	6.89	3	2	7.56	1
**32**	420.65	3.33	6.53	2	2	8.43	1
**33**	420.65	3.33	6.53	2	2	8.43	1
**34**	470.73	1.67	6.81	4	0	4.87	1
**35**	472.75	2.16	7.15	4	0	3.86	1
**36**	381.64	−2.06	4.81	3	1	4.60	0
**37**	227.39	0.73	3.74	2	2	10.30	0
**38**	422.63	1.47	1.47	3	1	8.50	0
**39**	443.45	4.81	4.82	5	3	18.18	0
**40**	285.34	2.93	3.83	1	2	11.36	0

All physicochemical properties, including molecular weight (MW), log *P*, log *D*_5.5_, hydrogen bond acceptors (HBA), hydrogen bond donors (HBD) and percentage polar surface areas (%PSA), were calculated using Instant JChem (version 6.03). %PSA defined as topological PSA/van der Waals surface area ×100.

The results (Table 1 and Figure 6) suggested that the majority of isolated natural products obeyed Lipinski’s rule-of-five in terms of MW < 500 Da (92%), log *P* < 5 (87.5%), HBA < 10 (97.5%) and HBD < 5 (97.5%), although we have previously reported that log *D*_5.5_ is a more useful parameter to classify the lipophilicity of ionisable natural products [14]. 

### 2.6. Chemical Clustering

Cluster analysis of the isolated natural products was undertaken to identify congeneric chemical series. Canvas (version 1.6) was used to calculate 32-bit linear, path-based chemical fingerprints using Daylight invariant atom types [41]. To enhance the discriminating power of the chemical fingerprint, bits present in more than 95% or less than 5% of compounds were discarded.

**Figure 7:**
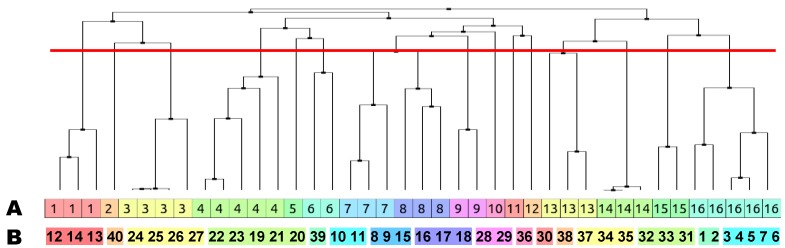
Dendrogram of chemical clustering of 40 isolated natural products with individual members colored by: (**A**) cluster number and (**B**) structure numbers. The red line indicates the clustering level.

Hierarchical clustering was then performed using the average distance between all inter-cluster pairs of a Tanimoto similarity matrix calculated from the fingerprints. Although the Kelley criterion [41] suggested 11 clusters were statistically optimal, the merging distance was manually decreased until each resulting cluster presented more structurally homogeneous groupings. The hierarchical clustering combined the 40 individual natural products into 11 chemical classes and 5 singletons as indicated in Figure 7 (with individual member structures memberships shown in red text on Figure 5). 

The structural classes identified by the hierarchical clustering showed an excellent correlation with the partitioning of observed antitrypanosomal activity [1,2,3,4,5]. The most active chemical classes identified were the pyridoacridine alkaloids (**12**–**14**) in cluster 1, the cinnamoyl amino acids (**10**–**11**) in cluster 6, the aryl amines (**8**–**9**) in cluster 7, and the cyclic peroxides (**1**–**2**) in clusters 16.

### 2.7. ChemGPS-NP Analysis

Rather than consider each physicochemical property in isolation, we were also interested in how these properties combine to influence the drug-likeness of the isolated natural products. ChemGPS-NP is a “global map” representing the limits of biologically relevant chemical space where the individual coordinates are *t*-scores from principal component analysis (PCA) using 35 descriptors calculated from 1779 chemical structures [42]. While ChemGPS-NP is comprised of eight coordinate dimensions (principal components, PCs), the four most significant PCs explain 77% of the variance in the training data and can be interpreted as representing broad physical properties such as size, shape, and polarizability (PC1); aromatic and conjugation related properties (PC2); lipophilicity, polarity, and H-bond capacity (PC3); and flexibility and rigidity (PC4). 

The online ChemGPS-NP WEB service [42,43] was used to examine the distribution in chemical space of the 16 cluster centroids identified in Figure 7 relative to a selection of 1491 FDA approved small molecule drugs (MW < 500) sourced from DrugBank [44,45,46].

**Figure 8:**
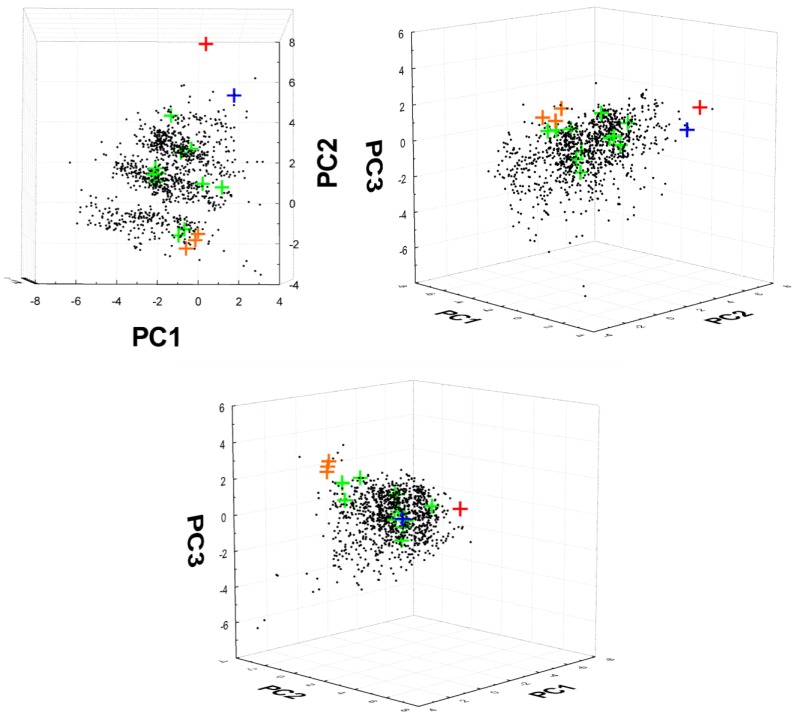
Distribution of 16 cluster centroids (crosses) and FDA approved small molecule drugs (black dots) in ChemGPS-NP chemical space defined by the first three principle components (PC): PC1 representing broad physical properties such as size, shape, and polarizability; PC2 representing aromatic and conjugation related properties; PC3 representing lipophilicity, polarity, and H-bond capacity. The drug-like cluster centroids are shown in green. Cluster centroids representing the lepadins (**31**–**33**), petrosine alkaloids (**34**–**35**), amino hydrocarbons (**36**–**37**) are shown in orange, pyrrole alkaloid (**39**) in blue, and the pyridoacridine alkaloids (**12**–**14**) in red.

The analysis (Figure 8) illustrated that almost all cluster centroids were positioned within drug-like chemical space when visualized using the most significant ChemGPS-NP coordinates in three dimensions. Only one cluster, the pyridoacridine alkaloids in cluster 1, was located well outside the cloud of points representing the FDA-approved small molecule drugs (Figure 8, red cross). Unsurprisingly, a closer examination revealed that these compounds (**12**–**14**) exhibit much higher aromaticity/conjugation (extreme PC2 value) than normally observed in drugs. Clusters centroids representing the lepadins (**31**–**33**), petrosine alkaloids (**34**–**35**), amino hydrocarbons (**36**–**37**) are located at the very limits of the FDA approved drug cloud since they contain too many rotatable bonds and exhibit high log *P*, although the log *D*_5.5_ values shown in Table 1 were significantly lower in each case (Figure 8, orange crosses). The pyrrole alkaloid (**39**) singleton shown as a blue cross in Figure 8, was positioned at the upper limits of drug-like chemical space due to high aromaticity, log *P*, and molecular weight. Eleven clusters of compounds (clusters 2, 3, 4, 6, 7, 8, 9, 11, 12, 15 and 16) with drug-like physicochemical properties were identified as desirable starting points for further chemical and biological investigations, they were cyclic peroxides (**1**–**7**) (cluster 15 and 16), aryl amines (**8**–**9**) (cluster 7), cinnamoyl amino acids (**10**–**11**) (cluster 6), bromotyrosine derivatives (**15**–**18**) (clusters 7 and 8), makaluvamine alkaloids (**19**–**23**) (cluster 4), aplysinopsins (**24**–**27**) (cluster 3), hymenialdisine alkaloids (**28**–**30**) (cluster 9 and 11), a purine derivative (**38**) (cluster 12), and a β-carboline alkaloid (**40**) (cluster 2). 

## 3. Experimental Section

### 3.1. General Experimental Procedures

All solvents used for SPE, HPLC, and MS were Lab-Scan HPLC grade, and the H_2_O was Millipore Milli-Q PF filtered. Dimethyl sulfoxide (DMSO, 99.9%) and TFA (99%) were obtained from Fluka. Oasis HLB was obtained from Waters. Commercially available Oasis HLB cartridges (400 mg) were employed to generate the fraction library. HPLC separations were performed on a Phenomenex C_18_ Monolithic HPLC column (4.6 mm × 100 mm). 

A Bio-line orbital shaker was used for large-scale extractions. Alltech Davisil 40–60 μm 60 Å C_18_ bonded silica was used for pre-adsorption work. A Waters 600 pump equipped with a Waters 996 PDA detector and a Waters 717 autosampler were used for HPLC. A Gilson 215 liquid handler (5 mL syringe, 200 µL Rheodyne sample loop) was used for injection and fraction collection. The liquid handler was controlled by Gilson 735 software (version 6.00). A ThermoElectron C_18_ Betasil 5 μm 143 Å column (21.2 mm × 150 mm) was used for semi-preparative HPLC separations [11]. 

### 3.2. Animal Material

The marine samples were collected in Queensland and Tasmania, Australia, by SCUBA diving. Samples were kept frozen prior to freeze-drying and extraction. Voucher samples have been lodged at the Queensland Museum, Brisbane, Australia.

### 3.3. Construction of Fraction Library

Freeze-dried and ground marine invertebrate samples (300 mg) were extracted with *n*-hexane (7 mL). The *n*-hexane extract was discarded, and each sample then extracted with 80:20 CH_2_Cl_2_/MeOH (7 mL) and dried. A second extract using MeOH (13 mL) was collected in the same glass test tube and dried to afford the crude extract. Further extraction and purification protocols refer to previous publication [14]. 

### 3.4. Extraction and Isolation

The freeze-dried and ground marine organism (10 g) was poured into a conical flask (1 L), *n*-hexane (250 mL) was added and the flask was shaken at 200 rpm for 2 h. The *n*-hexane extract was filtered under gravity then discarded. CH_2_Cl_2_:MeOH (4:1, 250 mL) was added to the de-fatted sponge material in the conical flask and shaken at 200 rpm for 2 h. The resulting extract was filtered under gravity, and set aside. MeOH (250 mL) was added and the MeOH/marine organism mixture was shaken for a further 2 h at 200 rpm. Following gravity filtration the biota was extracted with another volume of MeOH (250 mL), while being shaken at 200 rpm for 16 h. All CH_2_Cl_2_/MeOH extracts were combined and dried under reduced pressure to yield crude extracts. A portion of this material (1.0 g) was pre-adsorbed to C_18_-bonded silica (1 g) then packed into a stainless steel cartridge (10 × 30 mm) that was subsequently attached to a C_18_ semi preparative HPLC column. Isocratic HPLC conditions of 90% H_2_O (0.1% TFA)/10% MeOH (0.1% TFA) were initially employed for the first 10 min, then a linear gradient to 100% MeOH (0.1% TFA) was run over 40 min, followed by isocratic conditions of MeOH (0.1% TFA) for a further 10 min, all at a flow rate of 9 mL/min. Sixty fractions (60 × 1 min) were collected every minute from the start of the HPLC run. The fractions of interest were analyzed by LC-MS and bioassay, further purifications were carried out predominantly by reverse-phase C_18_ HPLC eluting with gradient MeOH/H_2_O containing 0.1% TFA to yield pure natural products. 

### 3.5. Cheminformatics

1137 reference drugs were obtained by filtering the DrugBank small molecule dataset (DRUG_GROUPS like “approved”, not like “withdrawn”, not like “nutraceutical”; Molecular Weight <500 Da; no metal ions).

Canvas (version 1.6) [41] was used to calculate 32-bit linear fingerprints with Daylight invariant atom types, excluding bits set in less than 5% or more than 95% of molecules. Hierarchical clustering using average cluster linkage was performed using a Tanimoto similarity matrix calculated from the chemical fingerprints. The clustering level was manually adjusted (0.7569 merging distance) and the 16 resulting cluster centroids exported for ChemGPS-NP analysis.

ChemGPS-NP coordinates for all structures were calculated using the online web service.

## 4. Conclusions

A combined Lipinski’s rule-of-five, cluster analysis and ChemGPS-NP principle component analysis of 40 marine natural products led to the identification of 16 chemical clusters, with 11 clusters positioned within drug-like chemical space. The results demonstrated that the initial enrichment of the screening library based on physicochemical profiling can translate into isolation of natural products with desirable physicochemical properties for oral bioavailability. The combined Lipinski’s rule-of-five, chemical clustering and ChemGPS-NP analysis can be employed as a beneficial strategy for the prioritization of active marine natural products for further investigation. 
